# Short term effectiveness and experiences of a peer guided web-based self-management intervention for young adults with juvenile idiopathic arthritis

**DOI:** 10.1186/s12969-017-0201-1

**Published:** 2017-10-13

**Authors:** Judy Ammerlaan, Harmieke van Os-Medendorp, Nienke de Boer-Nijhof, Lieske Scholtus, Aike A. Kruize, Philomine van Pelt, Berent Prakken, Hans Bijlsma

**Affiliations:** 1 Department Rheumatology & Clinical Immunology HPN D02.244, University Medical Center Utrecht, PO Box 85090, 3508 GA Utrecht, the Netherlands; 2 Department Dermatology and Allergology HPN D02.244, University Medical Center Utrecht, PO Box 85090, 3508 GA Utrecht, the Netherlands; 3 Department of Rheumatology & Clinical Immunology HPN F02.127, University Medical Center Utrecht, PO Box 85090, 3508 GA Utrecht, the Netherlands; 4 Department of Pediatric Immunology, Erasmus MC, Department of Rheumatology, University Medical Center Utrecht, PO Box 2040, Room Nb 852, 3000 GA Rotterdam, Netherlands; 5 Department of Pediatric Immunology, University Medical Center Utrecht, PO Box 85090, 3508 AB Utrecht, Netherlands; 6 Department Rheumatology & Clinical Immunology, HPN D02.244, University Medical Center Utrecht, PO Box 85500, 3500 GA Utrecht, the Netherlands

**Keywords:** Young adults, Juvenile idiopathic arthritis, Peer-guided, Self-management, Web-based intervention, Effectiveness, Short-term, Experiences

## Abstract

**Background:**

A web-based self-management intervention guided by peer-trainers was developed to support young adults’ self-management in coping with Juvenile Idiopathic Arthritis (JIA). To investigate its effectiveness, a randomized controlled trial (RCT) was conducted. In addition, the content of the chat and participants’ goals were studied to identify underlying processes.

**Methods:**

An RCT with a six-month follow up period was conducted among 72 young adults with JIA, aged between 16 and 25 years old, randomly assigned to the intervention or to the usual care control group. After 24 weeks, in both groups 24 participants completed all measurements. Intentions to treat analyses were carried out by means of linear mixed models for longitudinal measurements. With self-efficacy as primary outcome, self-management, disease activity, quality of life, absenteeism of school/work, health care medication use and adherence to the intervention were studied. The participants’ goals, personal achievements, interactions on the chat, and their appreciation of the intervention were analyzed using thematic analyses.

**Results:**

No significant differences were found on self-efficacy, quality of life, and self-management between the participants of the control group and the intervention group. In the intervention group, modeling and sharing experiences were the most recognized themes. Fifty-five goals were formulated and divided into the following categories: *improvement and maintaining balance*, *setting and recognizing boundaries*, *communicating* and *coping with incomprehension*. Adherence, appreciation of the own learning experience, and personal achievements were rated positively.

**Conclusion:**

The web-based intervention did not lead to an improvement of self-efficacy. However, additional qualitative analyses showed that the intervention was appreciated and valuable for the participants. More research is needed on how to measure the added value of this intervention compared to the usual care.

**Trial registration:**

Trial registration number NTR4679.

## Background

eHealth interventions are developed and offered more and more to patients with chronic diseases in order to improve their self-management [1–3]. Since young adults are one of the most active groups of internet users, eHealth interventions like portals and self-management support programs may also be promising options for patients with Juvenile Idiopathic Arthritis (JIA) [1, 2, 4–12]. Furthermore, traditional health care services do not always suit the needs and problems of young adults, aged between 16 and 25 years, with JIA [8–13]. In the Western world, the reported incident of JIA varies between 1 to 22 cases per 100.000 children, with a prevalence of 8 to 150 cases per 100.000 [13]. The disease and its treatment put extensive demands on children and young adults as well as on their parents, due to precisely-scheduled daily medication requirements, regular physical exercise regimes and regular visits to the pediatrician or rheumatologist [14, 15]. Most of these young adults still experience problems well into their adult years with on-going medical treatment and significant disability [12–14]. Like other young adults, young adults with JIA have to develop their own identity and independence, but for them, the path towards adulthood is a lot bumpier [11, 15, 16]. As they become more independent, they will gradually become more responsible for their own illness and its treatment; they will become self-managers. Enhanced self-management may prevent disease exacerbation and facilitate a successful transition into adult care [2, 9, 17] and can be best described as ‘the individual’s ability to manage the symptoms, treatment, physical and psychological consequences and life style changes, inherent to living with a chronic illness as JIA’ [18]. At the University Medical Center Utrecht (UMC Utrecht), a web-based self-management intervention was developed in close cooperation with young adults, the Dutch Youth Network for young people with arthritis (YouthRwell.com), and both multidisciplinary teams of the child and adult rheumatology department [19].

The web-based intervention, which is called “Challenge your arthritis”, www.reumauitgedaagd.nl/jongeren (URL in Dutch) is based on the self-efficacy theory of Bandura [20] where self-efficacy stands for the confidence in one’s own ability to achieve indented results, such as self-management. Self-management may be enhanced by increasing self-efficacy [18] through practicing, observing others (modeling), meeting beliefs of others and by interpretation of physiological and emotional status. All these elements are embedded in the web-based intervention.In order to display pro-active behavior one needs to set personal goals prior to starting the program [21]. Therefore, goal-setting is a crucial element of the intervention. The intervention is led by young peer trainers in the age range of 20–30 years who suffer from arthritis themselves. They are recruited and selected through assessments and interviews conducted by the Dutch Rheumatism Patient League (DRPL). Finally, the peer leader was trained through a train-the-trainer educational program by the UMC Utrecht and a professional coaching organization (Work21.nl). This program consisted of following the training as a participant, learning about the different themes, and studying training skills.

The first draft of the intervention was evaluated on items of perceived usefulness, perceived ease of use, user acceptance and adherence in 12 young adults and 4 peer trainers, and appeared to be feasible, especially in dealing with problems in daily life [19]. Although the intervention is thought to be a practical aid in health practices, a randomized controlled trial (RCT) was conducted to investigate its effectiveness on self-efficacy, self-management, and quality of life. In addition, thematic analysis [22] within the intervention group was carried out to explore the interaction in the Chat, the goals the participants set, and the appreciation of the intervention itself.

## Methods

### Design

A RCT, with a six-month follow up period was conducted among young adults with JIA, who were being treated at the transition outpatient clinics of the UMC Utrecht and the Erasmus Hospital Rotterdam in the Netherlands. Participants of the intervention group were given access to the web-based self-management intervention. Qualitative thematic content analyses [22] were then used to explore the interactions in the Chat, the content of the formulated goals, participants’ personal achievements regarding their goals, and the appreciation of the intervention itself. The control group was a waiting list group, who were granted access to the web-based intervention after 6 months.

### Participants

Young patients, aged between 16 and 25 years, were eligible to participate if they were a) diagnosed with JIA b) abled to speak and read Dutch, c) had access to the internet and a mobile phone, and d) hadn’t participated in a self-management intervention before. Participants were recruited by their pediatrician/rheumatologist at the transition outpatient clinic. After information, informed consent had been obtained – for the participants < 18 years, also from their parents - the participants were asked to fill in online baseline measurements, prior to randomization.

### Randomization

Because the intervention was group-based with six participants per group, randomization was carried out each time 12 participants were included in the study. Since more women are diagnosed with a rheumatic disease compared to men, stratified block, randomization for the factor ‘gender’ to equally divide ‘men’ and ‘women’ among both groups, using a computerized intervention with an automated process, was conducted with no interference from the investigator. After randomization, the participants (and the parents of the participants < 18 years) received the allocated condition by email and post.

### Intervention and control group

Both the control group and the intervention group received the usual care, based on medical guidelines [23], consisting of a 3 monthly visit to the transition outpatient clinic where medical problems, questions, and treatment plans were discussed and an assessment of the disease activity was carried out by the pediatrician/rheumatologist or the transition nurse. Furthermore, the transition nurse also focused on problems and questions on dealing with the consequences of having JIA and coordinated the process of transition. Both control and intervention group were allowed to use information presented on the website jong-en-reuma.nl (in Dutch). This website contains information about medical issues and themes such as dealing with the consequences of having a rheumatic disease, feeling depressed, exercise, work and study, relationships and intimacy [6].

### The intervention group

In addition to the usual care, the intervention group started within 1 month, after randomization, with the web-based self-management intervention. Challenge your arthritis consists of password-protected, interactive web-based self-management instruction with three components: a Chat section, home exercises and a discussion board. Once a week, the group (six participants, two trainers) had a planned group Chat for a maximum of 90 min. Within the Chat, the weekly theme was clarified, goals were set and the participants were allowed to practice, ask questions, give and receive feedback, play a game or watch a real life story video based on the weekly theme. The weekly themes were based on the six themes of the intervention and outlined in Table 1. The home exercises were also discussed and evaluated. After the Chat, participants were allowed to work through the intervention at any time at home and do the exercises (1 hour per week). The home exercises consisted, for example, of reading information, watching a video, or practicing communication skills at school or work. The content of the home exercises was related to the weekly theme or their personal goals. The Chat and exercises, both created with input of young adults themselves, were supported by short videos in which young adults with a rheumatic diseases or a member of the multidisciplinary team spoke about their experiences with JIA. In addition, a discussion board was used by trainers and participants to offer encouragement and share tips.

**Table 1:** Themes and content of the web-based self-management intervention Challenge your arthritis

Themes	Contents
Are you a self-manager?	• introducing yourself, get in touch with the group • what do you (want to) know about your disease? • capacities and talents • goal setting and action planning
Friends, family and communication	• communication strategies • communication with school, friends, work, parents, health care providers • giving and receiving feedback • setting boundaries
Feeling blue	• receiving therapies (treatment, medication) • pain, fatigue, feeling blue • asking and giving help • relaxation
Sport and exercises	• being active • motion and physical activity • maintain your plans
Relations and Intimacy	• body images and thoughts • having a relationship • having sex • thinking about kids, pregnancy, heredity
Having control over your life and arthritis	• evaluation of your personal goals; how to move on? • being responsible and making choices • celebration and saying goodbye

For 6 weeks, participants worked chronologically through the intervention using the six weekly themes (Table 1). On average, the total time investment for the intervention was 12 h in a 6-week-period per patient.

### Outcome measures

In this study quantitative and qualitative outcome measures were collected online with questionnaires, text messages, self-reported by participants. All outcome measures were collected at baseline, 3 and 6 months after randomization. Demographic and disease related variables were collected at baseline, supplemented with data from the medical record. Internet skills were assessed with a short questionnaire on general and health related use of the internet, similar to the questionnaire used in the study of van Pelt [7] on the use and relevance of health related internet sites by patients with JIA. A transcript of the interactions was available for thematic analyses of goals, personal achievements, and appreciation of the intervention.

### Quantitative outcomes

#### Primary outcome measurement

The Dutch Arthritis Self-efficacy Scale (Dutch- ASES) [24] was used to measure self-efficacy as a determinant of self-management behavior. This online questionnaire was translated from the German-ASES which showed an internal consistency of 0.90 in a similar study population. The translation process included repeated forward-backward translation, an expert group opinion, and testing of the Dutch-ASES in the patient group (*n* = 12) to ensure content validity. The questionnaire contains eight items. For each item, respondents were asked to indicate on a scale from 1 (very unconfident) to 10 (highly confident) how confident they felt in bringing a situation to a good outcome. The mean score of the eight items was calculated where a higher score indicated a higher degree of self-efficacy.

#### Secondary outcomes measurement

The following secondary quantitative outcomes were assessed: self-management, quality of life, medication use, health care use, absenteeism of school or work, personal learning experiences, and adherence with the intervention.

The Dutch Health Education Impact Questionnaire (Dutch heiQ) [25] was used to measure self-management related outcomes. This online questionnaire was translated, culturally adapted and validated in a validation study [25] among adults suffering from a chronic illness, like arthritis. Results from this study showed an internal consistency of 0.67–0.85 on the eight scales, comparable with the original English version [26] and was found to have robust psychometric properties, and to be user friendly and well understood. This Dutch heiQ consists of 40 questions, divided into eight independent scales which cover eight self-management domains: ‘Positive and active engagement in life’, ‘Health directed activity’, ‘Skills and technique acquisition’, ‘Constructive attitudes and approaches’, ‘Self-monitoring and insight’, ‘Health service navigation’, ‘Social integration and support’, and a reversed scale, ‘Emotional distress’*.* Each scale is calculated a mean score (min 1, max 4). A higher mean score indicates a higher degree of self-management in each domain, on each scale.

The Dutch Consensus Health Assessment Questionnaire Disability Index (HAQ-DI) [27] was assessed to measure the first dimension of quality of life (QoL); physical functioning. The second dimension of QoL was a combination of 4 patient reported outcomes: pain, fatigue, general well-being, and disease activity. This dimension was assessed with a Numerical Rating Scale (NRS) [28] from ‘0’ to ‘10’ (the higher the score, the more pain, fatigue, or disease activity and the worse general well-being). For 3 days in a row, on a fixed time, participants were asked to send their responses via a text message.

Medication and health care use were assessed by analyzing the medical patient record.

Medication was divided in the following categories: Disease Modifying Anti Rheumatic Drug (DMARD), Non Steroid Anti Inflammatory Drug (NSAID), Biologicals and use of paracetamol. Health care use was operationalized by counting the number of consultations to the pediatrician/rheumatologist/transition nurse and day-care center at the hospital. Absenteeism of school or work was assessed with one question (days of absenteeism of school last month, due to JIA) by a text message on one fixed moment.

At the end of the intervention, participants of the intervention group were asked to indicate with a score from 0 to 10 on a NRS how they rated their own learning experience. Also, in this group, adherence with the web-based intervention was measured by the researcher by counting the amount of total participation in the weekly Chat.

### Qualitative outcomes

Interaction in the Chat, goals, personal achievements with regard to their goals, and appreciation of the intervention were explored using thematic analyses [22] within the intervention group. With thematic content analyses, relevant written fragments/transcripts are first categorized in main themes, related to the goals of the study and the intervention, and further categorized into sub-themes [29]. Results were discussed on several occasions and differences were discussed until consensus was reached.

### Statistical analysis

#### Quantitative analyses

The consolidated Standards of Reporting Trials (CONSORT) statement [30] was used to present the results of this study. Quantitative data was entered into a SPSS data base. Based on the theoretical fundament of the intervention, it was hypothesized that participants of the intervention group would have a better result on self-efficacy, compared with the usual care control group participants. Sample size calculation, based on a previous study of Niedermann et al. [31], suggested that 72 patients were required to find a difference between both groups of 1.29 (*sd* 1.6) on self-efficacy, with a power of 80% and alpha of .05 and, an estimated loss of 30% .

Demographic variables and absenteeism of school/work, frequency of health care use and medication use were presented using descriptive analyses and frequency scores. Adherence of the intervention group in the Chat, and the indication score of their own learning was counted and displayed with a frequency score. Linear mixed models for longitudinal measurements were used to determine the effects on self-efficacy, self-management related outcomes and the effects on QoL scores for physical activity, pain, well-being, and fatigue and disease activity. Linear mixed models is a statistical model especially used for repeated, longitudinal measures [32] and is a proven, reliable statistical procedure which deals with missing values.

Fixed effects for group, time and group versus time interaction were included in the model.

#### Qualitative analyses

All transcripts of the Chats were inserted into the program Nvivo (QSR International Pty LTD Version 10). To describe the interaction in the Chat, the transcripts were analyzed, using codes like *modeling, mastering, verbal persuasion, goals, sharing experiences, emotional attitude* and *appreciation*, derived from the self-efficacy theory and the elements of the intervention.

Relevant fragments within the transcripts related to goals, personal achievements and appreciation of the intervention were categorized independently by two members of the research group (JA and NdBN). Results were discussed until consensus was reached.

## Results

**Fig. 1:**
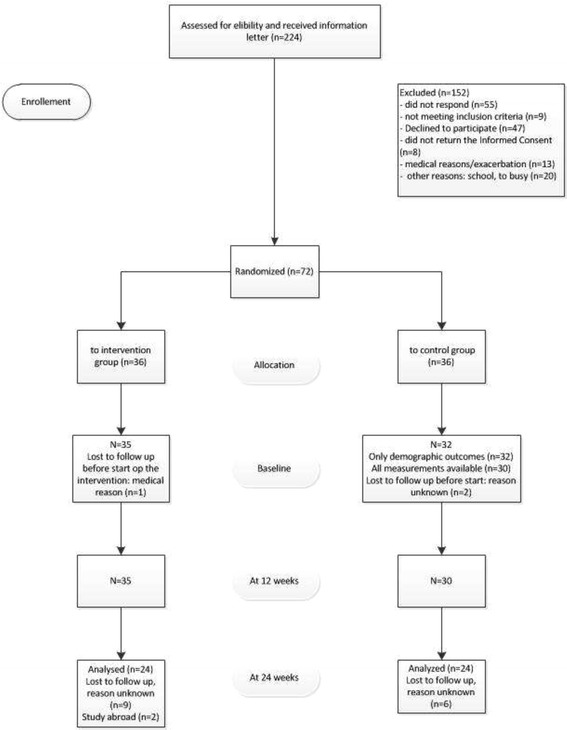
Flowchart of the participants

A group of 224 young adults with JIA were found to be eligible to participate in this study and were invited. 152 were excluded of which the largest group (55/152) was unreachable by phone to give additional information or to check inclusion criteria. 47/152 declined to participate in the study after being informed that the intervention lasted 6 weeks and participation in all Chat sessions was mandatory (see Fig. 1). Finally, 72 participants with a mean age of 19 years old, most of them female (88%) were randomized, equally divided into the intervention and the control group (see Table 2). Five participants (4 of the control group and 1 of the intervention group) did not fill in the baseline questionnaire, with unknown reasons. At the follow-up, after 6 months, 24 participants (67%) of the intervention and 24 (67%) of the control group filled in all questionnaires. There were no significant differences between the intervention and control group on baseline on demographic variables or internet-skills. Furthermore, there were no differences between the completers and non-completers on demographic and illness-related data on baseline data.

**Table 2:** Baseline characteristics of the participants

Baseline characteristics Intervention versus Control			
Characteristic	Total group (*n* = 67)	Intervention group (*n* = 35)	Control group (*n* = 32)
Gender – female n (%)	59 (88%)	29 (83%)	30 (94%)
Age, mean years (SD)	19.1 (2.7)	19.2 (2.7)	19.1 (2.9)
Education level completed n (%)			
Primary education^a^	15 (22%)	10 (29%)	5 (16%)
Intermediate vocational education^b^	35 (52%)	17 (49%)	18 (56%)
Bachelor or University	17 (25%)	8 (23%)	9 (28%)
Civil status n (%)			
Living at home with parents	53 (79%)	26 (74%)	27 (84%)
Living independently	14 (21%)	9 (26%)	5 (16%)
Marital status n (%)			
Single	61 (91%)	32 (91%)	29 (91%)
Living together	3 (5%)	1 (2%)	2 (6%)
Married	3 (5%)	2 (6%)	1 (3%)
Diagnosed disease n (%)			
Oligo-articular JIA	14 (21%)	8 (23%)	6 (19%)
Poly-articular JIA	24 (36%)	9 (26%)	15 (47%)
Systemic JIA	8 (12%)	6 (17%)	2 (6%)
Other	21 (31%)	12 (34%)	9 (28%)
Duration of the disease (years diagnosed, mean, SD)	10.9 (6.4)	10.1 (6,3)	11.8 (6.5)
Frequency of internet use n(%)			
(Almost) daily	62 (93%)	31 (89%)	31 (97%)
Several times a week	3 (5%)	2 (6%)	1 (3%)
Few times a week	1 (2%)	1 (3%)	
Rarely	1 (2%)	1 (3%)	
Purpose use internet			
Search health info n(%)			
Not	3 (5%)	2 (6%)	1 (3%)
Yes, one single time	30 (45%)	18 (51%)	12 (38%)
Yes, sometimes	28 (28%)	11 (31%)	17 (53%)
Frequent	6 (9%)	4 (11%)	2 (6%)
Forum visit health/arthritis			
Not	47 (70%)	23 (66%)	24 (75%)
Yes, one single time	12 (18%)	7 (20%)	5 (16%)
Yes, sometimes	6 (9%)	4 (11%)	2 (6%)
Frequent	2 (3%)	1 (3%)	1 (3%)
Chat/e-mail with someone with arthritis			
Not	55 (82%)	27 (77%)	28 (88%)
Yes, one single time	5 (7%)	4 (11%)	1 (3%)
Yes, sometimes	2 (3%)	2 (6%)	
Frequent	5 (8%)	2 (6%)	3 (9%)
Visit weblog patient			
Not	55 (82%)	30 (86%)	25 (78%)
Yes, one single time	9 (13%)	2 (6%)	7 (22%)
Yes, sometimes	2 (3%)	2 (6%)	
Frequent	1 (2%)	1 (3%)	
Relevance internet in relation			
disease, Scale 1–10 (mean) (*SD*)	3.4 (2.5)	3.1 (2.7)	3.7 (2.1)
peer support, Scale 1–10 (mean) *(SD*)	3.0 (2.5)	3.1 (2.6)	3.0 (2.5)

*SD* standard deviation

^a^Lower vocational education, lower general secondary education

^b^higher general secondary education, pre-university education

### Primary outcome: Self-efficacy

No significant differences between the intervention and control group were found on self-efficacy at 3 and 6 months (*p* = 0.136) (see Tables 3 and 4).

**Table 3:** Results linear mixed models for Dutch-ASES, HAQ-DI, pain, well-being, fatigue, disease-activity and heiQ

	Source	F	Sig.
Dutch Arthritis Self-efficacy Scale	Intercept	1322.47	0
	T	0.51	0.61
	group	0.17	0.68
	T * group	2.07	0.14
Dutch Consensus Health Assessment Questionnaire (HAQ-DI)	Intercept	68.32	0
	T	0.21	0.81
	group	0.01	0.93
	T * group	1.26	0.29
Perceived pain	Intercept	141.02	0
	T	1.34	0.27
	group	0.28	0.60
	T * group	0.25	0.78
Perceived well-being	Intercept	386.33	0
	T	0.88	0.42
	group	0.73	0.40
	T * group	2.50	0.09
Perceived fatigue	Intercept	401.63	0
	T	1.63	0.21
	group	0.43	0.51
	T * group	0.21	0.81
Perceived disease activity	Intercept	146.02	0
	T	1.45	0.24
	group	0.16	0.69
	T * group	2.12	0.13
Health Education Impact Questionnaire (heiQ)			
Health directed activity	Intercept	2011.89	0
	T	0.33	0.72
	group	1.15	0.29
	T * group	1.66	0.20
Positive and active engagement in life	Intercept	3936.64	0
	T	0.05	0.95
	group	1.61	0.21
	T * group	0.61	0.55
Emotional distress	Intercept	909.73	0
	T	0.21	0.81
	group	0.02	0.90
	T * group	0.43	0.66
Self-monitoring and insight	Intercept	6216.50	0
	T	1.39	0.26
	group	0.83	0.37
	T * group	0.92	0.40
Constructive attitude and approaches	Intercept	3941.58	0
	T	0.17	0.85
	group	0.08	0.78
	T * group	0.91	0.41
Skills and technique acquisition	Intercept	2534.79	0
	T	1.06	0.35
	group	0.70	0.41
	T * group	1.26	0.29
Social integration and support	Intercept	2328.77	0
	T	1.22	0.31
	group	0.50	0.48
	T * group	1.15	0.32
Health service navigation	Intercept	2907.22	0
	T	0.25	0.78
	group	2.29	0.14
	T * group	1.83	0.17

*F* statistic, *T* Time, T *group interaction time and group, *Sig* significance

**Table 4:** Results on Dutch Arthritis Self-efficacy Scale (Dutch-ASES), Pain, Wellbeing, Fatigue, Disease Activity, HAQ-DI, HeiQ, based on linear mixed models

	Intervention group	Control group
	Mean (95% CI)	Mean (95% CI)
Dutch-Arthritis Self-efficacy Scale^a^		
T0	6.67 (6.11–7.23)	6.99 (6.38–7.59)
T1	6.67 (6.41–7.54)	6.80 (6.17–7.40)
T2	6.51 (5.88–7.15)	6.84 (6.19–7.50)
Perceived Pain^b^		
T0	3.19 (2.39–3.98)	3.06 (2.23–3.89)
T1	3.59 (2.69–4.50)	3.11 (2.15–4.07)
T2	3.65 (2.76–4.55)	3.38 (2.44–4.32)
Perceived Wellbeing^b^		
T0	4.59 (3.82–5.36)	4.24 (3.44–5.03)
T1	4.93 (4.25–5.62)	4.03 (3.29–4.76)
T2	4.12 (3.28–4.96)	4.24 (3.36–5.12)
Perceived Fatigue^b^		
T0	5.36 (4.55–6.16)	5.05 (4.21–5.89)
T1	5.45 (4.66–6.23)	4.95 (4.11–5.79)
T2	4.94 (4.09–5.79)	4.75 (3.86–5.64)
Perceived Disease Activity^b^		
T0	3.31 (2.57–4.06)	3.01 (2.23–3.79)
T1	3.70 (2.81–4.58)	3.02 (2.08–3.97)
T2	3.42 (2.49–4.36)	3.73 (2.75–4.71)
Dutch Consensus Health Assessment Questionnaire (HAQ-DI)^c^		
T0	0.66 (0.46–0.87)	0.62 (0.40–0.85)
T1	0.67 (0.44–0.90)	0.59 (0.34–0.83)
T2	0.62 (0.37–0.87)	0.70 (0.44–0.97)
Health Education Impact Questionnaire (heiQ)^d^		
Health directed activity		
T0	2.98 (2.78–3.18)	2.94 (2.72–3.16)
T1	3.13 (2.92–3.33)	2.86 (2.64–3.07)
T2	3.08 (2.85–3.32)	2.96 (2.72–3.21)
Positive and active engagement in life		
T0	3.25 (3.12–3.38)	3.18 (3.04–3.32)
T1	3.30 (3.13–3.47)	3.11 (2.93–3.29)
T2	3.28 (3.09–3.47)	3.16 (2.96–3.36)
Emotional distress		
T0	2.00 (1.84–2.16)	1.97 (1.79–2.14)
T1	1.93 (1.73–2.12)	1.99 (1.78–2.20)
T2	1.99 (1.74–2.24)	2.01 (1.75–2.27)
Selfmonitoring and insight		
T0	2.97 (2.84–3.11)	3.01 (2.87–3.16)
T1	3.17 (3.01–3.32)	3.04 (2.88–3.20)
T2	3.11 (2.96–3.25)	2.99 (2.84–3.13)
Constuctive attitude and approaches		
T0	3.29 (3.14–3.43)	3.33 (3.17–3.49)
T1	3.34 (3.16–3.51)	3.23 (3.05–3.41)
T2	3.28 (3.10–3.47)	3.26 (3.06–3.45)
Skills and technique acquisition		
T0	2.83 (2.65–3.00)	2.85 (2.66–3.04)
T1	3.00 (2.81–3.20)	2.81 (2.61–3.01)
T2	3.02 (2.80–3.23)	2.90 (2.68–3.11)
Social integration and support		
T0	3.10 (2.91–3.29)	3.11 (2.90–3.31)
T1	3.20 (3.01–3.39)	3.10 (2.90–3.30)
T2	3.15 (2.93–3.37)	2.97 (2.74–3.20)
Health service navigation		
T0	3.16 (3.01–3.31)	3.12 (2.96–3.29)
T1	3.28 (3.09–3.46)	3.03 (2.84–3.23)
T2	3.31 (3.10–3.52)	3.06 (2.84–3.28)

*T0* baseline, *T1* 3 months after baseline, *T2* 6 months after baseline

^a^measured on a scale from 1 to 10 (1 (very unconfident) to 10 (highly confident)

^b^measured on a NRS scale from 0 to 10 (the higher the score, the more pain, fatigue or disease activity and the worse general well-being)

^c^measured on a scale from 0 to 3 (0 (no effort) to 3 (impossible)

^d^measured on a scale from 1 to 4 (1 (not all true) to 4 (exactly true)

### Secondary outcomes

We found no significant differences between the two groups on the secondary outcomes. Results on health care use showed that participants of both groups have had a consultation with a transition nurse. Considering the consultations with the pediatrician or rheumatologist, the median score of the intervention group was 3 (min 0 – max 28), where the median score of the control group was 3.5 (min 0 – max 28). No differences were found between the control and intervention group on absenteeism of school or work. The use of biologicals at baseline was higher in the control group (31,3%) compared to the intervention group (11,4%) (see Table 5).

**Table 5:** Absenteeism, health care use and medication use

	Intervention group		Control group	
		n		n
Absenteeism of school/work (days, previous month)				
T1: median (min-max)^a^	0.00 (0–10)	30	0.00(0–10)	27
T2: median (min-max)^b^	0.00 (0–20)	25	0.00(0–30)	24
Frequency of health use (whole period)				
Consultation Pediatrician/rheumatologist, median (min-max)	3 (0–11)		3.5 (0–28)	
Consultation Transition clinical nurse, median (min-max)	1 (0–7)		0 (0–10)	
Day-care center, median (min-max)	0 (0–11)		0 (0–8)	
Medication use				
Non Steroid Anti-rheumatic Drug (NSAID)				
T0: n (%)	12(34.3%)	35	9(28.1%)	32
T1: n (%)	15(42.9%)	35	10(31.3%)	32
T2: n (%)	12(34.3%)	35	8(25.0%)	32
Disease Modifying Anti Rheumatic Drug (DMARD)				
T0: n (%)	19(54.3%)	35	16(50.0%)	32
T1: n (%)	19(54.3%)	35	15(46.9%)	32
T2: n (%)	16(45.7%)	35	11(34.4%)	32
Biologicals				
T0: n (%)	4(11.4%)	35	10(31.3%)	32
T1: n (%)	7(20%)	35	10(31.3%)	32
T2: n (%)	7(20%)	35	8(25%)	32
Medication to reduce pain (paracetamol)				
T0: n (%)	6(17.1%)	35	5(15.6%)	32
T1: n (%)	2(5.7%)	35	4(12.5%)	32
T2: n (%)	2(5.7%)	35	5(15.6%)	32

^a^Mann-Whitney U (Asymp.Sig (2-tailed)) = 313.50 (0.09)

^b^Mann-Whitney U (Asymp.Sig (2-tailed)) = 284.00 (0.69)

### Qualitative outcomes

Within the Chat, modeling and sharing experiences were recognized as the most frequently expressed interaction. In addition, support and encouragement were also given. In total, 55 individual goals were formulated by the 32 participants at the start. However, a further 112 personal goals were added during the intervention due to personal reflection or increased awareness. Most formulated goals were related to ‘improve and maintain balance during the day’, ‘setting and recognizing boundaries’, ‘improving communication with others’, and ‘coping with incomprehension’. Additionally, goals like ‘increasing knowledge about the disease’, ‘coping with emotional (fear, uncertainly) and psychical consequences (pain, fatigue)’ and ‘fitting treatment advice for daily life’ were formulated.

A total of 145 personal achievements were categorized into: *‘gaining insight and awareness’*, *‘making determined, informed choices’, ‘personal influence’* ‘*understanding’* and *‘new goals for the future’.*

Their own learning performance was rated with a mean score of 7.1 (min 6, max 8.5; on a scale 0–10). The four participants with a self-appointed grade of 6, did not always relate the learned personal achievement to their originally defined goals.

Some of the participants were surprised by the aim and structure of the intervention, 33 of the 35 participants who started the intervention stayed adherent and were active in the Chat and performing their exercises. Two participants stopped due to medical reasons and school activities. All participants appreciated the intervention, especially the input of the trainers. Some remarks were made about the time of the weekly Chats (start time, duration) and the initial pace of the Chat (too slow). Due to technical problems (slow download speed), not every video led to the outcome that was hoped for.

## Discussion

In this RCT on the effectiveness of the web-based self-management intervention Challenge your arthritis among young adults with JIA, no significant differences were found between the intervention group and the control group on self-efficacy, quality of life (QoL), and self-management. On the other hand, participants of the intervention group rated their personal achievements within the intervention positively, and adherence and appreciation of their own learning experience was high.

There are some possible explanations for not identifying significant improvements on the patient reported quantitative measurements. Firstly, both control and intervention groups registered relatively high baseline scores on the domains of self-efficacy, self-management and QoL, so there was little room for improvement. This could have been influenced by the setting of the hospitals were the participants of this study were recruited. Both university hospitals have a special transition outpatient clinic and a multidisciplinary team and are known as large, tertiary care centers in the Netherlands where the focus already lies on guidance towards self-management. Also, both groups were under treatment by a pediatrician, rheumatologist, or transition nurse, receiving medical treatment, and rated themselves relatively low on disease-activity. Secondly, although we chose as outcome measure ‘self-efficacy’, based on the theoretical fundament of the web-based intervention, one might question if this outcome is acceptable to measure the concept of self-management, considering the age and needs of the young adult. To measure self-efficacy, we used the Dutch-ASES, a questionnaire, developed for adult patients, suffering from a rheumatic disease. As we know from studies on transitional care [9–12] but also from studies on ‘growing up with a chronic disease’ [4, 8, 11], young adults experience other difficulties in managing arthritis in daily life and have other needs compared to the adult group. So it is possible that not only the concept but also the language and aim of the adult self-efficacy scale did not fit with this age group and was therefore not sensitive to change. Clearly, interchangeability of a child and adult questionnaire, measuring the same concept, can be problematic. The absence of questionnaires measuring the concept of self-management from the perspective of young adult’s group was also recognized in the study of van Pelt [33]. More research is needed on how to measure the concept of self-management and the meaning of the concept, based on the views of young adults.

Finally, a response shift could have influenced the outcomes of this study. A response shift is defined as ‘a change in the meaning of one’s self evaluation of a target construct as a result of recalibration’ [34] and is recognized as an influencing factor to the outcomes of educational and behavioral interventions. Relatively high scores at baseline could be explained by unawareness of already existing self-management related behavior, since analyses of Chats revealed that participants gained awareness, increased influence on their own situation, and made more informed decisions. It is unknown to what extent the process of awareness had also taken place as a consequence of the usual care both groups received.

The personal goals, set by the participants of the intervention group, reflected the aim of the intervention, and the needs and real-life issues of young adults with JIA, which are known from literature on these subjects [4, 10, 11, 16]. During the intervention, personal goals were added which can be seen as a result of increased awareness of needs. In a study on health care transition in rheumatology [12], awareness is recognized as an important factor towards autonomy. Obtaining and increasing autonomy is, in this study, seen as an important psycho-social developmental task of adolescence. Analyses of the interactions in the Chat revealed that many experiences and strategies were shared, and support and encouragement were mutually exchanged. Sharing experiences and strategies to solve problems, and exchanging support and encouragement are recognized as active coping styles and predictors for psychological adjustment in young adults [35]. Stimulating the use of these styles may prevent developmental problems in psycho-social functioning.

The aim of the intervention was to enhance the young adults’ self-management in coping with JIA. The qualitative results expose that the intervention ‘delivers what it has to deliver’ and suggest that the intervention fits the needs of this group. These qualitative results, as well as other studies on needs assessments among young adults with JIA [4, 8, 9], point out the importance of paying attention to the complexity of managing medical needs, together with developmental aspects and the drive towards independence. The web-based intervention is appreciated by young adults and can be a valuable aid for both young adults and health care professionals in order to support and improve self-management. The intervention could be valuable in delivering eHealth instead of face-to-face self-management support for young patients who live far from the hospital. Further research is needed to determine which patient benefits most from online self-management support. Furthermore, some adjustments have to be made. Future participants should be better informed about the aim and the structure of the intervention. Additionally, support should be provided to identify the personal needs and goals they want to achieve. This can prevent potential dropout and provide an early indication of those patients who will benefit the most. Contact with the peer trainer before registering for the intervention can be helpful in this process and can improve the effectiveness of the intervention.

There are some limitations to our study that should be mentioned. Firstly, we recruited participants from two large tertiary centres in the Netherlands. Some participants expressed that they participated because they had a good relationship with their doctor. This could have affected their results positively and therefore generalizing the results may not be entirely possible. Secondly, we chose patient reported outcomes (PROs) to capture the young patients’ perspectives on the effectiveness of the intervention. Measuring and describing clinical outcomes with, for example, the JADAS (for patients < 18 years old) or the DAS (for patients > 18 years old) could have been a valuable contribution, but as discussed before, interchangeability of a child and adult questionnaire is problematic, Thirdly, the qualitative results are only representative of the young adults who participated in the intervention group; we did not study these outcomes in the control group. Finally, although the predetermined calculated number of patients was included, there was some drop-out in the follow-up leading to a small sample size at the end of the study. However, mixed-method analyses were used in which drop-outs were taken into account [36].

## Conclusions

In our study on the effectiveness of the web-based intervention Challenge your arthritis for young adults with JIA, we did not find improvement of self-efficacy, self-management, and quality of life. However the intervention was regarded to be a valuable and appreciated aid to influence an active coping style by sharing experiences, enhancing social support, and increasing autonomy and goal-setting behavior. More research is needed on how to measure the added value of this intervention/self-management in this group, and on what meaning young adults themselves give to the concept of self-management.

## Abbreviations

CONSORT: The Consolidated Standards of Reporting Trials
DMARD: Disease Modifying Anti Rheumatic Drug
Dutch-ASES: Dutch-Arthritis Self-Efficacy Scale
HAQ-DI: Dutch Consensus Health Assessment Questionnaire Disability Index
HeiQ: Health Education Impact Questionnaire
JIA: Juvenile Idiopathic Arthritis
NRS: Numerical Rating Scale
NSAID: Non Steroid Anti Inflammatory Drug
QoL: Quality of life
RCT: Randomized Controlled Trial
SPSS: Statistical Package for the Social Sciences
